# Development and Experimental Verification of an Ergonomic Backpack

**DOI:** 10.1155/2020/1437126

**Published:** 2020-05-15

**Authors:** Mohamed Z. Ramadan, Sultan N. Al-Tayyar

**Affiliations:** Industrial Engineering Department, King Saud University, Riyadh 11421, Saudi Arabia

## Abstract

Carrying a heavy school backpack has extensively been reported as a prime cause of children's body strain. It is suggested that the load should not exceed 10 percent of the child's body weight; however, ensuring this requires continuous monitoring. The study explores how ergonomically designed school backpack based on the user anthropometric data (*n* = 280) and ergonomic parameters help reduce force concentration on shoulders and back. It provides a validation process of the developed prototype by experimental verification. The developed design was assessed in a comparison experiment with a commercially available local school backpack. An experimental study was used which recruited thirty healthy college students (aged 19 to 23 years). Two independent variables evaluated were school backpack type (developed backpack versus commercial one) and load levels as a percentage of body weight. Three load levels were employed 10%, 15%, and 20%. These variables were measured on the responses: bag comfort scale and the percent of maximum voluntary contraction (%MVC) of six muscles (right and left of erector spine, right and left of external abdominal oblique muscle, and right and left of trapezius). The developed backpack provided astonishing performance at levels of 15% and 20% of body weight in terms of subjective measure and electromyography (EMG) responses. It also showed that increasing the carried weight more than 10% result in reducing activity on the erector spinal muscles, while it increases on abdominal oblique muscles. The developed backpack design confirmed the efficiency of its bases by distributing the carried weight among the trunk through side pockets, attached to the body through two upper and lower straps. It helped the body to distribute the carried weight and avoid concentrating pressure on specific areas.

## 1. Introduction

Carrying school backpack is a common practice observed across all stages of academic life. School students begin to carry their bags from the first year in the school until last year at the University. Several studies report this activity as a risk to the student's health [1–3]. Although the relationship between the use of a schoolbag and back pain is uncertain, it has attracted the attention of various medical and safety societies concerning the safe use of school bags [4]. Back injuries are one of the most expensive and eternal health problems as it can lead to further complications in the future [5]. Other reported problems include changes in spinal curvature and forward lean of the head and trunk [1–5].

Studies denote weight as prime and critical elements of backpack carriage [6–8]. Hong and Cheung [9] study noted that nine and 10-year old primary school children carry weights of more than 15% of their body weights, which alert trunk flexion to a large extent and is detrimental for their health. Sahli et al. [10] pointed out critical postural changes among students who carry schoolbag more than 15% of their body weights. Additionally, it causes considerable changes in trunk inclination when students carry a weight of more than 20% of their body weights [11]. Other effects of increased trunk lean forward [12–15], increased cardiorespiratory responses [16], increased loads on lumbar intervertebral discs [17], and increased foot-ground pressures [18, 19]. The detrimental effects of school bag exercise weight extend to lessen students' walking efficiency [20–22] and decrease the walking time [23]. The apparent reason for the decline in walking time and speed is the effect of those who utilize backpack to take small steps (decreasing the length of step) and a smaller number of steps per minute and consume extra time on both feet when carried heavy schoolbags [24]. Although weight is viewed as the primary consideration of the schoolbag carriage, different factors such as the method of carriage, strap length, and time spent carrying school-bag among other factors influence the risk of musculoskeletal symptoms such as pain and discomfort in different body regions of primary school children [25–28].

Few studies emphasized that ergonomically designed backpack with an adaptation of anthropometric characteristics substantially decrease musculoskeletal disorders (MSDs) in students [29]. This stresses the vitality of opting for anthropometric in designing a backpack [30]. As primordial research mainly focused on the effectiveness of straps of backpacks, which proves that the contact pressure under the shoulder straps of the backpack significantly increased when carrying loads (10-30% of body weight) as per [31]. The contact pressure of the backpack strap on the underlying tissue is greater than the pressure threshold (approximately 30 mm Hg) to prevent skin flow [32]. Schoolbag straps often press shoulder in the front part, upright in the region over the brachial plexus, axillary artery, and vein [33]. If the pressure on these tissues continues, it may affect circulation and tactile feeling of hand/arm. Khan et al. [34] inferred that the pain differentiates in terms of area and frequency; however, the most widespread musculoskeletal disorders include shoulder (44.4%), followed by neck (29.6%), low back (23%), and upper back (3%), which are generally linked to the carrying of a backpack. Similar reports confirm that trapezius and erector spinae muscles were the most influenced parts altogether while wearing a school backpack [35, 36]. Park et al. [37] developed a wearable upper-body device to utilize some strategies that distribute the backpack loaded among shoulders and the pelvis. Recently, five studies reported that carrying and walking with backpack loads above 10% BW significantly alters trunk muscle activation levels [38–41].

Another study conducted in Saudi Arabia showed that pain generally experienced in the shoulder (70%) and back (18%) [42]. Earlier research in Saudi Arabia [38] endeavored to design a backpack by simulating life jacket, depicting positive results among different variables (such as muscular activities of the rectus abdominis, erector spinae, and cardiac cost). Several studies recommend that a backpack should be positioned high on the trunk to reduce its negative effect [36, 43]. Analysis of the previous studies has shown that the damaging effect of the backpack has been concentrated on the adult population (such as college students), identifying the risk factor or the back-placement position [31, 33]. Very few studies have provided a school backpack design. Therefore, the principle motivation behind this study is to develop a school backpack design for students in Saudi Arabia. It aims to aid in diminishing the possible adverse effects of wearing it, considering every one of these components to lessen the unwanted effects and help in developing the right of the school backpack. Therefore, the main objective of this research was to develop and to test an ergonomics school backpack design, which helps to eliminate the damaging effects of the carried load.

## 2. Materials and Methods

This study consists of two phases. The first phase was concerned with developing a school backpack design using users' anthropometric data, where the anthropometric measures were collected from a sample of 280 students. Then, the school backpack was developed and manufactured with specified design dimensions based on the collected anthropometric data. Also, the second phase was concerned with testing and validating the developed school backpack by comparing body muscle EMGs and subjective perception of the comfort for 30 participants between the newly designed backpack and commercial one.

The first phase was conducted to figure out the best school backpack design, including its dimensions. Consequently, some studies focused on positioning the carried weights on the back through testing shoulder strap tightness [44, 45]. Life jacket backpack, which developed and tested by Ramadan and Al-Shayea [38], provided positive results concerning the variables rectus abdominis muscular activity, erector spinae muscular activity, and cardiac cost. Also, Mosaad and Abdel-Aziem [46] confirmed that double-sided bags gave better results in terms of stability and reducing sagittal posture perversion of both head and neck for school students. Therefore, to get better results, the following was considered as bases for developing the new school backpack:

1. Distributing weight among other body parts instead of concentrating forces on shoulders and back only
2. Attach the weight as close as possible to the body by adding a strap in the lower and upper sides of the bag

**Figure 1:**
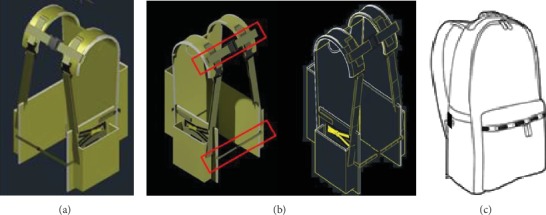
Two-side pockets attached with back pocket to form a new schoolbag. (a) Isometric of the developed schoolbag. (b) Two straps in the upper and lower sides in the front. (c) Commercial backpack.

The initial ergonomics designed backpack was drafted based on ergonomics features and standards, anthropometries and weight distributions, and with consideration of the user's requirements, preferences, and desires. While keeping the back pocket, two side pockets were added to the bag, as shown in Figure 1(a). Thus, three pockets in total employed in this developed backpack because the prospect idea of side pockets was assumed to disperse the weight from being concentrated on the back. Also, adding two pockets to both sides of the body known as “double-sided bag” or “BackTpack” was confirmed not to affect body balance as confirmed by Mosaad and Abdel-Aziem [46]. Also, two straps were added, as shown in Figure 1(b). One in the top and one in the middle of the front side of the back. The idea of those to straps is to keep the back adjoining the body and to give users flexibility for different body sizes. The top strap was expected to add another benefit, which is to keep the load on top of the back close to the body. Generally, the dimensions of the backpack consisted of the height, width, and thickness of the backpack with the width and length of the associated shoulder and waist straps. The difference between the developed backpack in this study and the recommended one in Ramadan and Al-Shayea [38] is the two-third of the carried weight that was distributed in the front of participant's chest as in Ramadan and Al-Shayea [38]; however, the distributed loads in this study were in both sides of the participant instead. And the rest third of the carried weight was in the back in both backpack designs.

### 2.1. Participants

#### 2.1.1. Participants Recruited in the First Phase

The first phase started with recruited 280 volunteer male college students from the university. The anthropometric measurements were adopted based on its acknowledgment as effective in the previous studies [29, 30, 38]. These measurements included sitting shoulder height, shoulder breadth, thigh clearance height, and body weight. Sitting shoulder height is defined as the vertical distance from the stool surface to the acromion (i.e., the shoulder's bony point). Shoulder breadth is defined as maximum horizontal breadth between shoulders, measured to the protuberances of the deltoid muscles. Thigh clearance height is defined as the vertical distance from the sitting surface to the highest point on the thigh. Measuring the width of the neck is one of the few measurements that require two tapes. The first tape left to fall on the shoulders in the front of the body. The second tape is used to measure the neck width. The measurement is then taken in a straight line between the two parts of the hanging tape of the neck. The weight is computed by weighting the student with a backpack and the weight of the student without carrying a backpack, so different can be computed. To measure participant anthropometric data, the followings were used:

1. Fiberglass tapes (Dean, 0-1500 mm)
2. Spreading caliper (0-600 mm)
3. Sliding caliper (Martin type with length 0-200 mm and depth of 0-50 mm)
4. Fixed anthropometry (0-2100 mm with straight probes and provided with curved measuring branches).

A calibrated balance scale (Seca 708, ±0.1 kg) was used to measure participants' weights. After collecting information, data was processed through normality testing, uniformity testing, homogeneity testing, and percentage data calculation. The designing of the backpack incepted by specifying the correct dimensions based on the data processing, following the creation of a prototype. Anthropometric measurements were gathered as shown in Table 1.

#### 2.1.2. Participants Recruited in the Second Phase

The second phase started with recruited the minimum number of participants that required in this study according to equation (1). This equation proposed by the International Standards Organization, ISO 15535 : 2012 “General requirements for establishing anthropometric databases,” with a 95% confidence interval for the 5th and 95th percentiles:

(1) $$\begin{array}{c} N\geq {\left(3.006\times \frac{cv}{\alpha }\right)}^{2} \end{array}$$

Where *N* is the required sample size numbers. *CV* is the coefficient of variation, and *α* is the percentage of the desired relative accuracy.

In this study, *α* = 5% relative accuracy is required for the 5^th^ and 95^th^ percentiles, whereas values of coefficient of variation *CV* are adapted from Ramadan and Al-Shayea [38]. As a result, the minimum sample size was 30 for weight parameter calculation.

Thirty healthy college male school students, with a mean age of 25.90 years and a standard deviation of 2.634, from the University, who were paid at a rate of Riyals 50 per hour. Selecting college students as a population for this study was based on their direct exposure to the problem. The participant anthropometric data are presented in Table 2, and all were healthy and had no history of back pain disorders or any other musculoskeletal injury. Any participant had a preexisting orthopedic problem, a history of the musculoskeletal disorder, injury to the arms, legs, spine, or a heart and/or lung problem or with an allergic reaction to any adhesives and gels put on the skin was excluded from the experiment.

These college students were the only ones approved by the Institutional Review Board IRB of the King Saud University (IRB Approval on Research Project No. E-18-3451), since they had the necessary health insurance (stipulated with the University). In addition, the new design should be implemented on students who have completed their musculoskeletal system. Despite our repeated attempts at also recruiting female students by distributing leaflets in the girls' section, only male participants volunteered for the experiment.

A visiting physician joint the experiment in order to ensure the validity of participants in this experiment, as requested by IRB. A written consent form approved by the Institutional Review Board (IRB) of the University, completed by each participant before participating in the experiment. The integral health history form was collected before they signed the consent form, and those forms were examined to classify students who are fit for participation in this study.

## 3. Experimental Protocol

### 3.1. Backpack Development

#### 3.1.1. Backpack Height

The height of the backpack is acquired from the student's sitting shoulder height. It is recommended that the maximum height of the backpack should not exceed ten centimeters from the fifth percentile of the student's sitting shoulder height. Then, as recommended in Mououdi et al. [30], Larisang [47], and Kristina and Amanda [48], backpack height is equal to subtraction of 2.5% of the thigh thickness from the 5% of the sitting shoulder height. Then, backpack height is equal to 5% of the sitting shoulder height minus 2.5% of the thigh thickness. This value is equivalent to 43 cm (e.g., 53.3-10.1). Since there are three pockets, and the length of the textbooks is ranged between 21.5 cm and 27 cm. Therefore, the backpack height is 30 cm.

#### 3.1.2. Backpack Width

The width of the backpack ought not to surpass the width of the student's body with the goal that the student can move all-around more freely. Thus, the width of the backpack is measured by determining the distance between the shoulders. The shoulder joint is simply the body width range. It is recommended that the maximum width of the backpack should not exceed the fifth percentile of the student's shoulder to shoulder distance. Then, as recommended in Mououdi et al. [30], Larisang [47], and Kristina and Amanda [48], the backpack width is equal to two-thirds of the 5% of the shoulder to shoulder length. Then, backpack width is equal to two-thirds of the 5% of the shoulder to shoulder length. This value is equivalent to 25 cm (e.g., 2/3 of the 37.3).

#### 3.1.3. Backpack Thickness

First, determine the maximum load of the backpack, which is based on 10% of the students' weights. 10% of the average weight of college students was 75.5 kg, as shown in Table 1. The volume of the backpack is acquired from the transformation of the most allowable backpack weight that has been determined earlier into backpack volume. Second, convert that weight to volume based on the following: 1 kg of paper materials (standard) can be converted to 832.8 cm^3^ [48], so later can be resolved the thickness of the backpack that can just suit the load as per the most allowable backpack weight. This volume is then analyzed to obtain the thickness of the backpack utilizing the volume equation (2).

(2) $$\begin{array}{c} Pocket\;volume=Total\;required\;volume/3=Height\times Width\times Thick\\ Pocket\;volume=total\;volume/3=Height\times Width\times Thick\\ =10/100*75.5*832.8/3=30*25*Thick \end{array}$$

Thickness ≈3 cm. 66% Allowance was added for materials that may has less specific gravity than paper. Then, thickness for each pocket = 5 cm.

#### 3.1.4. Shoulder Strap Width

By measuring the strap width, 90% of the strap width of the local market backpacks is 7.5 m. Considering that the wider the bag belt, the better the distribution of the load is, the shoulders are more uniform, and muscular areas do not work excessively. Shoulder strap width is equal to 95% of the neck width from 5% of the shoulder to shoulder length. Waist strap and chest strap lengths were designed adjustable from the 5th to the 95th percentile of body dimensions based on chest and waist circumferences [49]. Then, the shoulder strap width is equal to half of the subtraction of 97.5% of the neck width from 2.5% of the shoulder to shoulder length. The value would be 9.5 cm (e.g., (36-17)/2).

Finally, waist strap, chest strap, and shoulder strap were designed, which were adjustable from the 5th to the 95^th^ percentile of measurements, equivalent to 81.5 cm and 102.4 cm. In addition, the dimension of the height and width of the back pocket is equal to the width and height of each side pocket, respectively. Figures 1(a) and 1(b) present the developed backpack, which used cotton fabric material and mild steel frame.

### 3.2. Testing the Developed Backpack

The main objective here was to test the performance of the developed design of the schoolbag. Performance expressed in terms of schoolbag carrying effects on six muscles (right and left of the rectus abdominis muscular activities, erector spinae muscular activities, trapezius muscular activities) as an objective measure, and bag comfort scale as a subjective measure.

#### 3.2.1. Variables

*(1) Independent Variables*. Two school backpacks were used in this experiment commercial and designed one. The reason for using the two backpacks was to observe the difference (see Figures 1(a) and 1(c)). The commercial backpack, known to most people, is available in the local market based on two surveys. One survey was sent to 500 individuals randomly chosen across different colleges in the university, of which 269 individuals responded. The other survey was sent to the four leading book stores to determine which schoolbags the most were purchased. The results were used to determine which schoolbag is the most used in the community. The one used in the study is representative of most community use. It is classified as a classic look with durable pack features of polyester materials, external zippers with built-in rain hoods, a front zippered accessory pocket, a 15-inch laptop pocket, as well as padded shoulder straps and back panel. It includes an interior sleeve pocket and a single front pocket with key clip. Its dimensions are 18 by 12.25 by 5.5 inches.

Ten, fifteen, and twenty %BW were used in the experiment. Based on the studies conducted in Saudi Arabia, most students carried loads varying between 10% and 15% of their body weights [50–52]. Another 20% level used in several studies was added. This level is commonly used in such studies [12, 14, 38]. The load used here were the students' books with dissimilar weights, so were the sizes used and a group of books tied to each other to give more weight. As a result, book weight varies from 0.1 kg, 0.2 kg, 0.5 kg, 1 kg, 2 kg and 4 kg. During the experiment, the applied load was equally distributed among all the three pockets of the bag.

*(2) Dependent Variables*. The dependent variables were two. First is the percent of maximum voluntary contraction (% MVC) for right and left of erector spinae, right and left of rectus abdominis, right and left of trapezius muscle activities; and the second variable was bag comfort score.

#### 3.2.2. Experimental Design

A two-factor within-subject design was employed in which each participant was asked to walk at a normal pace as usual in a specific path around the laboratory (54-meter length) carrying both schoolbags containing three loads (one at a time and at an assigned specific level of weight). Muscular activity (e.g., right and left of erector spinae, right and left of external abdominis oblique muscle, and right and left of trapezius), bag comfort score of healthy college male was monitored and recorded as response variables. Every participant performed six runs carrying different loads in a random order to minimize learning effects.

#### 3.2.3. Equipment

Two schoolbags (developed and commercial ones available in the local market) were used to carry different loads. In this experiment, an eight-channel Bio-monitor ME6000, MT-ECG-1 preamplifier, and the Mega Win 3.0.1 software (Mega Electronics Ltd., Kuopio, Finland) were chosen to record physiological signals (six channels to record electromyography (sEMG) signals). Since the design of the experiment determined the loads need to be carried as 10, 15, and 20 present, different sets of calibration weight and calibration weight needed to be used in order to manage the accuracy of loads as participants' weights vary. The range of the calibration sets utilized varied from 100 g to 2.5 kg, where the 100 grams scale was the smallest unit used. Additionally, Matlab R2015 has brought into use to analyze EMG data. The anthropometric equipment that has been used in phase one was employed in this phase. Then, after weighing the participant, a calculator was used to compute the 10%, 15%, and 20% the of participant's body weight. Other materials and equipment included 70%-isopropyl-alcohol swabs, tissues, adhesive bandages, cotton squares, and Ag/AgCl solid adhesive pregelled electrodes for EMG signal acquisition (Ambu A/S, Denmark).

#### 3.2.4. Measured Responses

*(1) Electromyography (sEMG) Signal Responses*. To measure EMG potentials of an individual, electrodes were positioned on six muscles including trapezius muscles (left and right), erector spinae muscles (left and right), and rectus abdominis muscles (left and right). The Ag/AgCl solid adhesive pregelled electrodes were connected to 6-channel Biomonitor ME6000 (Mega Electronics Ltd, Kuopio, Finland). Rectus abdominis fiber direction was located at the level of the anterior superior iliac spine and 20 mm lateral to the midline. Erector spinae muscle sites were positioned at the level of the L4/L5 interspace and 20 mm lateral to the midline. Trapezius muscle originates on the spine and extends from T2 to T12 and inserts onto the spine of the scapula from the acromian process to its root. The skin surface was carefully prepared and cleaned by 70% alcohol before the electrodes were attached. The EMG signals were documented using the Mega Win software at a sampling rate of 1000 Hz. Low-frequency artifacts—such as the activity of neighboring muscles, respiration, and motion potentials—were removed applying a band-pass elliptic filter with a cut-off frequency range from 20 to 500 Hz. Then, a 50-Hz notch filter was used to remove the 50-Hz power line interference in the recorded EMG signals. The filtering process and extracting the signals of EMG values (in microvolts) have been done using MATLAB code.

*(2) Bag Comfort Rating*. This is defined as the sensation of a comfort carrying backpack during the student movement, and it contributes a worth-noting sensation of comparison among backpack types. Mills et al. [53] used a ranking scale for a comfortable sensation with a reliable measure. Participants in their experiment were enquired to rate the comfortable sensation from the most comfortable (rank 7) to noncomfortable (rank 0).

#### 3.2.5. Experimental Setup and Procedures

**Figure 2:**
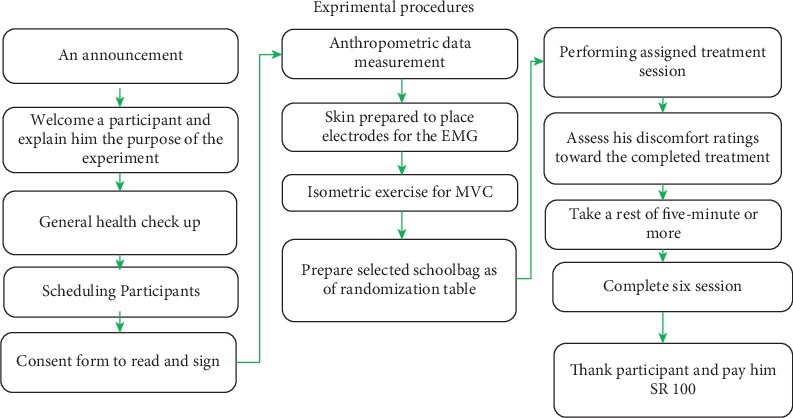
Experimental procedure (*n* = 30).

After the schoolbag was designed and made, the experimental data collection started. This section presents the detailed steps of the experimental procedure. The experimental procedure, as summarized in Figure 2, started after an announcement invitation, which was issued and distributed in the university in order to get adult participants.

In the annunciation, the participants were invited to participate in a pre- and six sessions comprising on from 45 to 120 minutes. Their weights were employed to calculate the levels of load employed for the experiment (10%, 15%, and 20%) as a percentage of the subject's body weight. Every participant has been explained in detail the purpose of the experiment, all steps involved, and the time required for each session and rest. After that, an opportunity was given to the participant to inquire about anything about the study without any hesitation. He was informed of his rights to stop or refuse his participation at any time in the experiment. Afterward, participants were screened for their health status and allergy to jell by a physician, which lasted for 4 minutes for each participant.

After welcoming participants, health checks, and explaining purpose and procedure, the participant was given a consent form to read and sign. Following it, anthropometric data were measured and recorded in addition to demographic data. These anthropometric data included the following: stature height, elbow-shoulder height, shoulder sitting height, elbow sitting height, hip breadth, shoulder breadth, elbow breadth, chest circumference, abdominal circumference, and weight. After that, the participant's skins were prepared using 70% isopropyl-alcohol swabs to place electrodes on their positions for the EMG. The EMG electrodes were fixed to the student's left and right of trapezius muscles, left and right of the erector spinae, and left and right of the rectus abdominis.

Participants were asked before participating in the experiment to perform the isometric exercise to the maximum possible extent for 3 seconds and instructed that this condition represented as a 100% EMG activity for the normalizing purpose. The %MVC for the erector spinae, trapezius, and abdominal muscles were recorded. Each participant was encouraged verbally during the %MVC tests to ensure they provided maximum exertion throughout the three seconds. Three repetitions were performed for each muscle with a one-minute recovery time between each repetition. The maximum of the three trials was considered as %MVC.

Muscle activities during maximum voluntary contraction (%MVC) of the six investigated muscles were recorded to be used for normalizing the muscle activities recorded during the experimental conditions. Hence, muscle activity data are expressed as a percentage of the maximum voluntary contraction (%MVC). The measurement procedures were standardized regarding body posture, verbal instructions, and encouragement [54, 55]. The principal investigator himself performed the measurements.

Before performing the assigned treatment session, the participant was asked to assess his comfort ratings across several parts of his body. After filling the schoolbag with measured weight as per the experiment randomization table, the researcher helped the participant to carry the schoolbag and ask him to walk carrying the bag around the lab perimeter for five minutes. The starting point was set for three meters from one edge of the laboratory corner.

A five-minute rest period was allowed between sessions to prevent fatigue. After the participant completed the assigned session, he was asked to assess his comfort ratings again toward the assigned treatment after carrying a schoolbag using descriptive scales. Once all sessions are completed, the electrode was removed. Lastly, a thank you note was presented to the participant.

#### 3.2.6. Analysis

The significance level threshold (type I error) for statistical analysis was set to 0.05. ANOVA for the repeated measures design was performed to test the main and interaction effects of schoolbags type and percentage of carried load on %MVC and backpack comfort rating. In a case of interaction is existed, a simple effect technique was employed. Design assumptions (normality, homogeneity of variance, and continuity of data) were examined to ensure the reliability of the statistical analysis results. Additionally, the effect size was calculated based on the partial eta-squared value (*η*2) to indicate the variance percentage independent variables that are attributable to a particular independent variable. The Friedman test was used to assess the differences between groups when the dependent variable being measured is ordinal such as subjective measure. In a case of finding a significant effect in a variable had several levels, Wilcoxon signed-rank tests were employed to differentiate among those levels. SPSS software (version 23) was used for analysis.

## 4. Results

After the normality test using Kolmogorov-Smirnov and Mauchly's sphericity test, which were conducted to confirm the homogeneity of variance, the computed results proved the normality of the data. In addition, the sphericity assumption was found violated. Hence, the degrees of freedom were corrected using Greenhouse-Geisser estimates of sphericity (*ε*) [56]. It showed that the backpack type had significantly violated the sphericity assumption, given the significant value below 0.05 (*W* = 1.0, *X*2 (0) = 0, *p* < 0.05). Thereby, the degrees of freedom (*df*) value for the main effect of the backpack type were corrected using Greenhouse-Geisser estimates of sphericity (*ε* = 1.0).

### 4.1. Bag Comfort Rating

**Figure 3:**
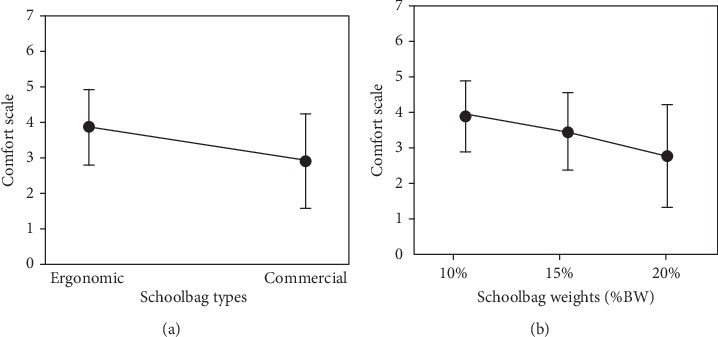
(a) Effect of schoolbag types on comfort scale. b) Effect of backpack load as %BW on comfort scale.

Participants who worn developed backpacks were more comfortable (*mean* = 3.887; *SD* = 1.05) than participants when worn the traditional ones (*mean* = 2.944; *SD* = 1.3); *p* < 0.0001, Wilcoxon signed-rank test (*Z* = −6.042), irrespective of the weight carried. Relatively, significant individual differences were computed, indicated by the high standard deviations. Also, the substantial effect of the carried load was found on the participant's comfort feeling (Friedman test, *χ*2 (2) = 62.227, *p* < 0.0001). Pairwise comparisons were used using Wilcoxon signed-rank tests to differentiate among the carried load levels. The results revealed an indirect relationship between carried load percent and comfortability (decreased at 20%), such as an increase in one causes a decrease in another increased (*mean* = 2.825; *SD* = 1.43) when compared to 15% (*mean* = 3.5; *SD* = 1.11) and 10% (*mean* = 3.922; *SD* = 1.0), (*Z* = −4.612),(*Z* = −5.942), *p* < 0.0001, *p* < 0.0001, respectively; as well as at 15% when compared to 10%, (*Z* = −3.754), *p* < 0.0001, as shown in Figure 3.

### 4.2. sEMG Responses

**Figure 4:**
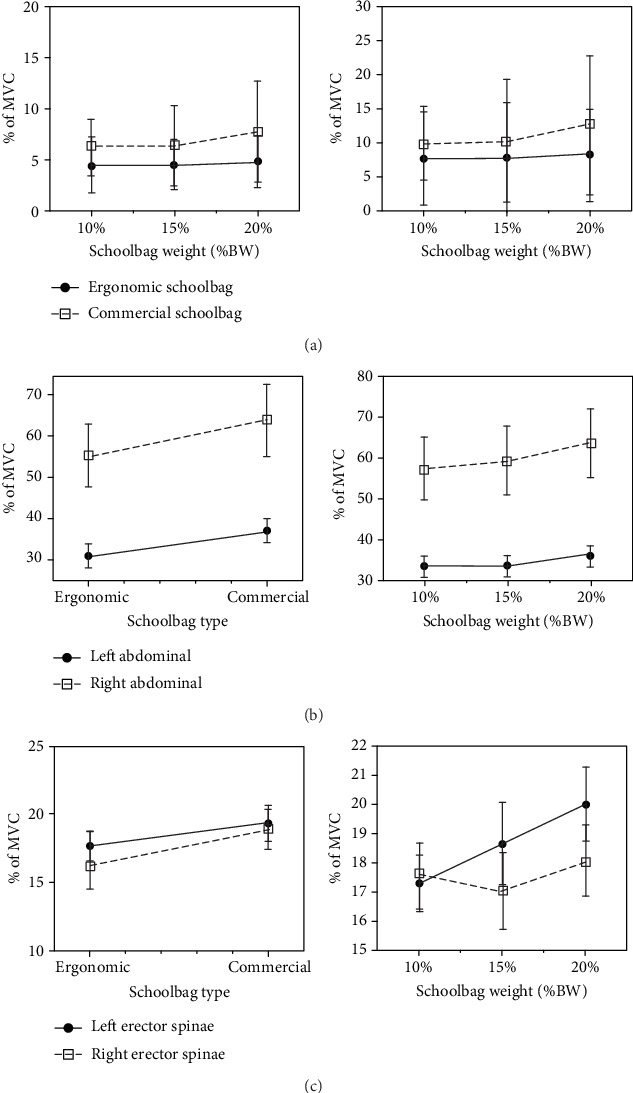
Effect of schoolbag type by weight interaction on (a) %MVC for both right and left trapeziuses. (b) %MVC of right and left abdominals. (c) %MVC of erector spinae muscles and the weight effect on left erector spinae.

Results for all muscles are demonstrated in Table 3. ANOVA analysis showed that %MVC for both right and left trapeziuses was substantially affected by the interaction between backpack type and load percentage [right trapezius *F* (1.743, 50.56) = 17.48, *p* < 0.001, *η*2 = 0.376] and [left trapezius *F* (1.957, 56.74) = 25.93, *p* < 0.001, *η*2 = 0.472].  Figure 4(a) shows the interaction effect between backpack type and percentage carried weight on %MVC for both trapeziuses. The results elaborated that the effect is symmetric on both trapeziuses. Nonetheless, it was noticed that the mean values were diverging, where the left trapezius always greater than the right trapezius. This might be because of the frequency of use of the right hand. Most people use their right hand so that minor distinctness might appear in the right muscle strengths.

For abdominal muscles, ANOVA analysis pointed out that backpack type significantly affected %MVC for both right and left abdominal muscles [right abdominal, *F* (1, 29) = 7.683, *p* < 0.01, *η*2 = 0.209] and [left abdominal, *F* (1, 29) = 9.043, *p* < 0.005, *η*2 = 0.238]. The backpack type significantly affected both measured muscular activities, which were substantially lower when students wore the ergonomic backpack than when wearing the commercial one for the right and left of abdominal muscles.

The analysis testified that backpack load significantly affected %MVC for both right and left abdominal [right abdominal, *F* (1.197, 34.706) = 4.166, *p* < 0.042, *η*2 = 0.126] and [left abdominal, *F* (1.955, 56.708) = 3.397, *p* < 0.041, *η*2 = 0.105].  Figure 4(b) verifies the backpack load effect on both abdominal muscles. The result showed that the developed bag exerts less effort into both abdominals. Moreover, it showed that regardless of the bag type, the effect of the weight lifted is moving increasingly with weight percentage increases. Only, at both abdominal muscles, wearing the schoolbag with 20% of body weights were higher stresses than when participants were wearing 10% of body weight, *p* < 0.023, and *p* < 0.05, for the right and left abdominal muscles, respectively.

For erector spinae muscles, ANOVA analysis exhibits that backpack type substantially affected %MVC for both right and left erector spinae [right erector spinae, *F* (1, 29) = 7.857, *p* < 0.009, *η*2 = 0.213] and [left erector spinae, *F* (1, 29) = 17.882, *p* < 0.0001, *η*2 = 0.381].  Figure 4(b) shows the backpack type effect on right and left erector spinae muscles.

Analysis escorts that load percentage significantly affected %MVC for left erector spinae [*F* (1.694, 49.113) = 8.754, *p* < 0.001, *η*2 = 0.232].  Figure 4(c) unveils the load percentage effect on the left erector spinae muscle, where 20% carried weight was significantly different from the 10% and 15%, *p* < 0.001 and *p* < 0.045, respectively. Also, the result shows that the developed bag exerts less effort into both erector spinae muscles, as shown in Figure 4(c).

## 5. Discussion and Conclusion

The study aimed to achieve two objectives: first, developing a backpack design in the light of studies done primordially. The second one was testing the developed backpack by comparing it with the commercial one. Preceding studies reported that shoulders and back were the main two areas that cause pain when an increased load on the back is carried [37, 50]. Similarly, another study [57] highlights the relationship between rectus abdominal muscle activity and load carried. In addition, a study [38] showed that backpack type significantly affects teethe cardiac cost and rectus abdominis muscular activity. The results showed that two independent variables were backpack type and carried load parentage. These variables were studied their effects on the measured responses such as bag comfort rate and %MVC of right and left of erector spinae, right and left of external abdominis oblique muscle, and right and left of trapezius.

Participants who wore developed backpacks felt more comfort than participants who wore the commercial ones regardless of the weight they carry. It was found that wearing the developed backpacks make the participants feeling more comfortable by 24.26% as compared to the commercial ones. The results revealed that as carried load percent increased, comfort feeling decreased by 23.89% at 20% when compared to 15% and by 38.83% when compared to 10%; as well as at 15% when compared to 10%, comfort score decreased by 12.06%.

On the other hand, the muscular activity of selected muscles (right and left trapezius, right and left erector spinae, and right and left abdominals) expressed in %MVC (maximum voluntary contraction) was measured. It caught a sight that the developed backpack reduced the muscular activity of trapezius, erector spinae, and abdominal compared to the commercial bag. Noticeably, the results of this study ushered that load percentage has a major influence on the muscular activity of left and right abdominals and right and left erector spinae. As expected, as the load percentage increased, muscular activity increased. This agrees with a study Al-Khabbaz et al. [58] saying that muscular activity was dependent on the weight of a backpack. Nonetheless, the vital particularity between the two bags appeared at more than 10% of body weight. The study showed a nonsignificant difference between the two bags at 10% of body weight. It could be a normal result as all bags approximately will have a similar effect on the body at 0% of body weight.

Notwithstanding, it was noticed that abdominal muscle activity increased as the load percentage increased. This was observed that abdominal muscles had more EMG activity than the rest of the selected muscles. It could be concluded that the human body adopts extra loads through transferring some forces to the abdominal muscles. This result agrees with Son [57]. Son [57] investigated the changes in muscle activities of the trunk and lower extremities as a result of changing of the backpack loads of 10, 15, and 20% of body weight. The author concluded that the muscular activity was remarkably skyrocketed only in the rectus abdominal muscle once the backpack loads of 10%, 15%, and 20% of body weight were implemented. Also, Son [57] in his study reported that muscular activity of the rectus abdominis muscles, erector spinae muscles, biceps femoris, and vastusmedialis muscles delineates that the muscular activity was tremendously taken height only in the rectus abdominis muscles when loads of 10%, 15%, and 20% of body weight were employed. Several studies highlighted students' complaints of the shoulder and back pains due to carrying schoolbags [1–8, 33].

Unfortunately, early and effective treatment should be provided as it can lead to severe and everlasting back issues. Standardizing a limit on the school bag load was repeatedly highlighted as a preventive solution. In contrast, different researches have tried to develop schoolbags designed ergonomically. In this study, a developed backpack design was developed to reduce the adverse effect of carrying a schoolbag on students. The developed backpack was designed and assessed practical. The assessment was based on a comparative approach in which a developed backpack was tested in comparison with a commercial one. Thirty university students aged between 20 and 30 participated in the experiment. Assessment of developed backpack was performed at different three levels of weight. Weight level expressed as a percentage of body weight. Therefore, three weight levels employed: 10%, 15%, and 20% of the participant's body weight. It was demonstrated that developed backpack proved better performance at levels of 15% and 20% of body weight in terms of subjective measure and EMG responses. However, at a level of 10%, it was not performing as desired. In reality, a large number of students carry more than 10% of their body weight, as demonstrated by various studies [3, 4, 14, 17]. However, the developed design confirmed that it is practical and adding a further step toward an optimum design.

The findings of the current study showed that increasing the carried weight of more than 10% while wearing a newly developed schoolbag would result in reducing trunk flexion when compared to the commercial one. This action could cause some relief on the erector spinae muscles. The design of a developed backpack might help the body to achieve this result through the side pockets in better functioning. Also, the contact area between the bag and the student body would be increased to reduce the pressure felt by students. It is also recommended to add more user-friendly delicate materials to be added to eliminate the probable size issue, which might add more stress because of friction. Softer material must be used in the inner sides of the side pocket to eliminate friction stress. These considerations were not possible in this study because of the limited time.

In this experiment, schoolbags weights were varying from participant to another, because the weight was expressed as a percentage of body weight. However, in reality, schoolbags mostly are equivalent in weight with minimal differences. Therefore, use three-fixed weight in future research could show very different results. Finally, the developed design confirmed the efficiency of its bases. Distributing weight among the whole body through side pockets, attach weight to the body through two upper and lower straps were confirmed in the new design. Moreover, the developed design confirmed that it helped the body to distribute weight and avoid concentrating pressure on specific areas. This is a vital factor in reducing the probable risk of carrying a schoolbag. Also, it enforced the pocket volume to be in a specific volume to keep the distributed weights as designed for.

The ergonomic measurements of the school backpack, including the backpack height and width, were determined for the students in a range of age 19 to 23 years by utilizing anthropometric dimensions. Many studies addressed the physiological and psychological effects and the imposed forces and stresses on the students' bodies caused by wearing backpacks [59]. In addition, several researchers tried to develop backpacks that fit and compatible with the students' bodies, though they did not improve the quality of life of the students. However, the paper presents an ergonomically acceptable design to achieve a reasonable success when the carried weight is distributed around the students' bodies considering their anthropometrical dimensions.

The findings of the study are limited, given its inclusion of only male participants and recruitment from a single university and region. To help expand the study scope, the same results are suggested to be replicated for the female participants and across different age groups; thus, the findings may not be generalizable to other genders or age groups. Accordingly, it is suggested that early educational interventions should be improved for minimizing the risk that emerges as a result of carrying the excessive load. Also, standardizing the schoolbag design can help reduce the complaints of back pain as a result of carrying bag load. In addition to the conclusions drawn regarding each previous hypothesis, this investigation did not study individuals who are obese and younger who carry backpacks. Therefore, those have higher instability and great changes in the posture leading to potentially at high-risk populations, and it should be investigated in future work to identify risks of injuries and falls. This study investigated the effects of short-term backpack load carriage; thus, the observations may not be applicable to long-term carriage, because trunk muscle activity may vary with fatigue and have significant effects on lumbar joint loads. Only three backpack loading conditions were evaluated, which might be a concern of generalizing the conclusions of this study. More detailed and larger-scale research is recommended for future study on the effect of backpack loads on the trunk and head inclinations, lumbar spine loading, gait analysis, cardiac effort (heart rate variability), respiration system, or at the joints of the lower limbs during walking biomechanically.

## Figures and Tables

**Table 1:** Anthropometric measurements of the 280 participants.

Anthropometric characteristics	Mean	SD	Min	Max	Percentiles				
					2.5	5	50	95	97.5
Age (years)	22.29	2.08	9	29	20	20	22	27	28
Weight (kg)	78.43	17.46	45	131	51.5	55	75.5	114	116.98
Stature height (cm)	173.68	6.03	157.1	191.7	163.6	164	174	184.5	185.5
Sitting shoulder height (cm)	65.71	6.84	47	98.7	49.6	53.3	66.1	74.9	76.0
Shoulder breadth-bideltoid (cm)	47.21	6.55	34	75.5	36.01	37.3	46.55	59.1	61.98
Thigh thickness (cm)	15.22	3.12	9	27.5	10.1	11	14.7	21	23
Neck width (cm)	11.32	2.39	7	18.4	7.5	7.9	11	15.78	17
Abdominal circumference (cm)	94.18	6.22	77.4	120.7	80.9	81.53	95	102.4	103.5

**Table 2:** Anthropometric measurements of the participants (*n* = 30).

Anthropometric data	Mean	SD	Range
Age (years)	25.90	2.64	30-20
Stature height (cm)	168.05	4.62	180-156
Weight (kg)	70.94	9.12	94-55
BMI of body weight (kg/m2)	25.11	2.98	32.53-18.37
Elbow shoulder height (cm)	46.29	1.42	49-44
Shoulder sitting height (cm)	65.45	2.67	70-60
Elbow sitting height (cm)	31.03	2.15	35-27
Hip breadth (cm)	36.47	2.65	43-32
Shoulder breadth (cm)	41.99	2.17	49-38
Elbow breadth (cm)	44.37	3.23	52.5-38
Chest circumference (cm)	90.06	6.32	108-78
Abdominal circumference (cm)	89.71	7.49	106-76

**Table 3:** Mean (SD) of the %MVC for all muscles.

Parameters	Mean (SD)						Statistics *P* (*η*2)		
Backpack type	Developed schoolbag			Commercial schoolbag			Backpack type (*η*2)	Load percentage (*η*2)	Interaction(*η*2)
Load percentage	10%	15%	20%	10%	15%	20%			
Right TR	4.55(2.73)	4.60(2.37)	4.89(2.55)	6.25(2.72)	6.42(3.87)	7.81(4.92)	0.013(0.193)∗	0.015(0.135)∗	0.0001(0.376)∗∗∗
Left TR	7.51(6.81)	7.85(8.09)	8.33(6.92)	9.98(5.38)	10.33(9.14)	12.65(10.09)	0.011(0.204)∗	0.017(0.130)∗	0.0001(0.472)∗∗∗
Right ES	17.57(8.69)	15.60(8.58)	15.15(9.19)	18.95(9.09)	18.18(7.72)	19.82(8.45)	0.009(0.213)∗∗	0.339(0.037)	0.079(0.088)
Left ES	19.08(7.52)	18.49(7.50)	15.76(4.96)	18.67(6.89)	18.67(8.43)	20.84(7.32)	0.0001(0.381)∗∗∗	0.001(0.232)∗∗	0.209(0.053)
Right Ab	51.05(34.5)	54.57(39.58)	61.79(48.40)	63.53(47.33)	63.96(50.39)	65.59(50.55)	0.01(0.209)∗	0.023(0.126)∗	0.115(0.078)
Left Ab	28.93(13.1)	31.04(14.59)	33.80(15.92)	37.76(17.57)	36.35(18)	37.97(16.40)	005(0.238)∗∗	0.041(0.105)∗	0.099(0.077)

∗Significant effect at *p* < 0.05; ∗∗significant effect at *p* < 0.005; ∗∗∗significant effect at *p* < 0.0005. TR: trapezius muscles; ES: erector spinae; Ab: abdominal muscles.

## Data Availability

The data used to support the findings of this study are included in the article.
